# Population genetic assessment of *Viburnum japonicum* in China using ddRAD-seq

**DOI:** 10.3389/fgene.2023.1150437

**Published:** 2023-06-01

**Authors:** Hong Zhu, Juan Liu, Meirong Gao, Chunlei Yue, Hepeng Li

**Affiliations:** Zhejiang Academy of Forestry, Hangzhou, China

**Keywords:** *Viburnum japonicum*, genetic diversity, population structure, DdRAD-seq, geographical isolation

## Abstract

*Viburnum japonicum* is a rare plant species and endemic to the coastal region of Eastern Asia with extremely small populations. Within mainland China, this species can be only found in narrow habitats of the northeast coastal islands of Zhejiang Province. However, there are scarce conservation genetic studies on *V. japonicum*, which has limited the effective conservation and management of this rare species. Here, 51 individuals in four natural populations covering the Chinese geographic range of the species were sampled to assess the genetic diversity and population structure. A total of 445,060 high-quality single nucleotide polymorphisms (SNPs) were identified using double digest restriction-site associated sequencing (ddRAD-seq). The overall average values of observed heterozygosity (*Ho*), expected heterozygosity (*He*), and average nucleotide diversity (*π*), were 0.2207, 0.2595, and 0.2741, respectively. The DFS-2 population exhibited the highest level of genetic diversity among all the populations. Genetic differentiation between populations was moderate (*F*_*ST*_ = 0.1425), and there was selfing between populations (*F*_*IS*_ = 0.1390, *S* = 24.52%). Of the total genetic variation, 52.9% was found among populations through AMOVA analysis. The Mantel test (*r* = 0.982, *p* = 0.030) combined with analyses of the Maximum Likelihood (ML) phylogenetic tree, ADMIXTURE, and principal component analysis (PCA), revealed that populations of *V. japonicum* were genetically segregated and significantly correlated with their geographical distribution. Our study demonstrated that *V. japonicum* maintained a medium level of genetic diversity and differentiation with a strong population structure, and the results were mainly affected by its island distribution pattern and self-crossing characteristics. These results provide insights into the genetic diversity and population history of *V. japonicum*, critical information for conserving and sustainably developing its genetic resources.

## 1 Introduction

Genetic diversity is a critical component of biodiversity, as it serves as an indicator of a species’ adaptability and changeability for change within its habitat (Reed and Frankham, 2003). During the long-term evolution of species, species need to maintain a high degree of genetic diversity, which facilitates the species’ potential for adaptive evolution (Wernberg, et al., 2018). Conducting genetic research on rare and endangered plant species (REPs) can aid in the conservation of these threatened resources. Such research could involve revealing the genetic diversity and distribution, genetic structure and differentiation of populations, as well as exploring the mechanisms that underpin the formation and maintenance of genetic diversity. By offering a scientific foundation for understanding the distribution and evolution of biodiversity, this research can provide vital data essential for the protection of REPs (Xu and Zang, 2023).

*Viburnum japonicum* (Thunb.) Sprengel, commonly known as Japanese viburnum, is an evergreen broad-leaved shrub in the family Adoxaceae. It can be easily distinguished from other recorded species of *Viburnum* by having characteristics such as rounded and evergreen shrubs, broad leaves, and glabrous texture. In particular, some studies have reported the leaf extraction of *V. japonicum* contained rich medical value. This indicates that the species has great potential for ornamental and economic use. In the past, the species was thought to be only naturally distributed on the coast of Japan (Mainly Honshu, Kyushu, and Ryukyu Islands). In 1994, *V. japonicum* was reported by Chinese plant taxonomists from the east coast of Zhejiang Province (Qiu, et al., 1994). By 2003, *V. japonicum* again occurred in South Korea (Gageo-do Island) (Hong and Im, 2003). Based on the field survey, most populations of *V. japonicum* survive in the island hillside forest, mixed wood forest, or rock pile, with an extremely small number. In addition, in recent years, along with the tourism development and economic activities of East Zhejiang islands, some wild habitats of *V. japonicum* have been affected to some extent. Thus, it was recognized as a plant species with extremely small populations (PSESP) and listed among Zhejiang Province’s Key Protected Wild Plants. Despite its endangered status and anthropogenic disturbance, genetic and genomic resources available for *V. japonicum* are scarce, compared to other *Viburnum* species. Previous research mainly focused on phytochemistry, resistance physiology, and breeding techniques. To the best of our knowledge, there was only one recent study relating to *V. japonicum* genetic diversity that has relied on the traditional molecular marker (inter-simple sequence repeat, ISSR). However, this traditional marker can provide only a small number of polymorphisms from a large sample collection. Next-generation technologies have revolutionized the depth of information we can get from a species’ genome (Janjua, et al., 2020). In the present study, to efficiently address the above drawback, we applied the ddRAD-seq approach to obtain genome-wide SNP makers for the population genetic analysis of naturally occurring *V. japonicum* populations along the Chinese coast. The study aims to answer the following questions as revealed by the detected SNP data: (1) The characterization of the pattern of genetic diversity and population structure of the four populations in China, (2) investigation of the gene flow and migration history, and (3) development of appropriate conservation and management strategies for long-term.

## 2 Materials and methods

### 2.1 Sampling collection and DNA extraction

**FIGURE 1:**
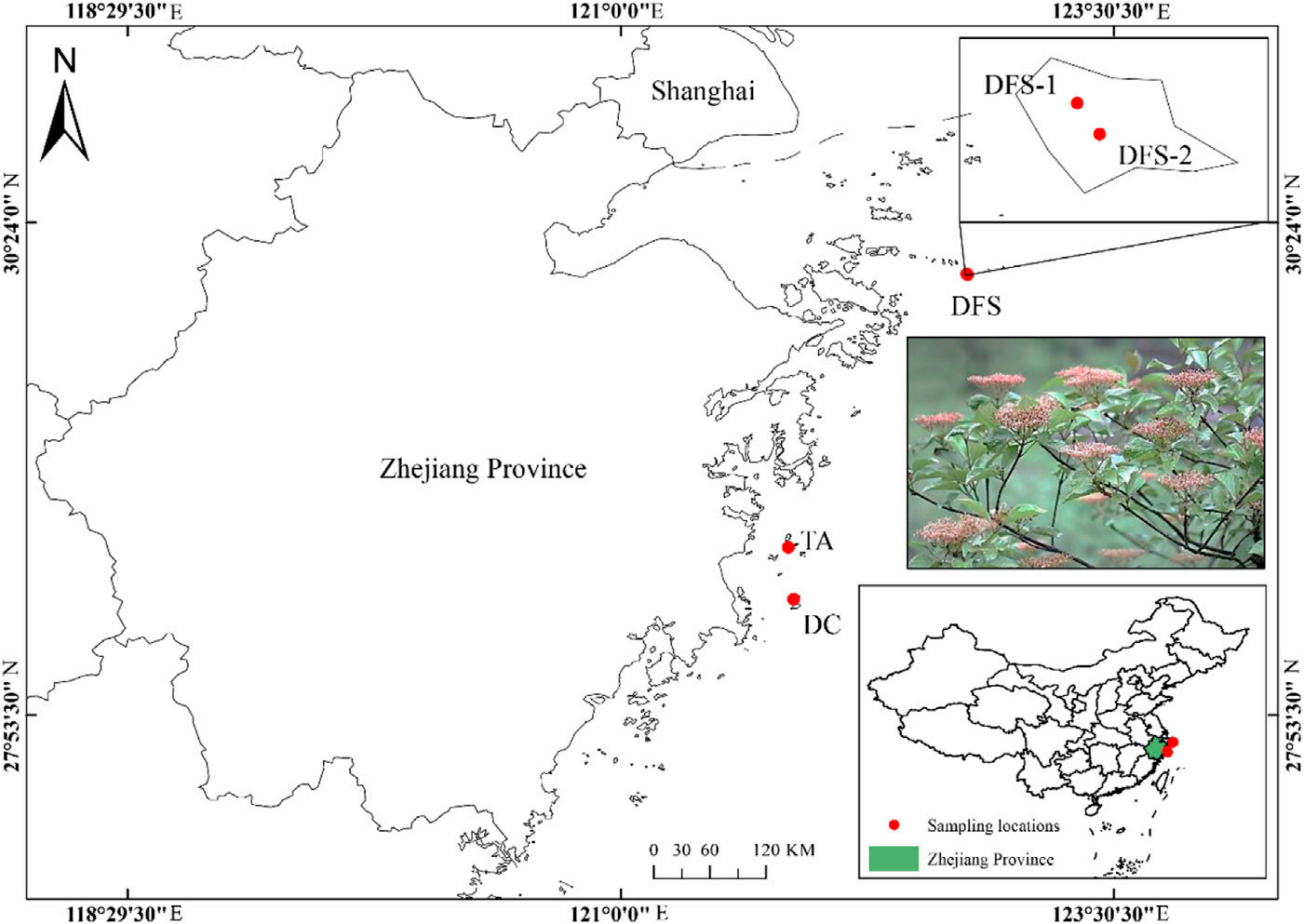
Sampling locations of four populations of *V. japonicum* cover nearly all known natural distributions across mainland China.

From January 2021 to October 2022, we conducted a collection of *V. japonicum* samples from nearly all known natural populations known to exist across mainland China. A total of 51 individuals from four natural populations were sampled and all the populations originated from islands within an altitudinal range from 45.84 to 230.81 m (DFS-1, DFS-2, DC, TA; Figure 1; Table 1). To preserve the species’ rarity in China, we chose not to publish detailed geographical coordinates. Fresh leaf tissues were collected from the field and preserved in plastic ziplock bags with silica gel desiccant until the DNA extractions. Genomic DNA extraction was performed with the dried leaf tissues using Plant Genomic DNA Kit (Tiangen Biotech, Beijing, China) following the manufacturer’s protocol. The quality was assessed via gel electrophoresis on 0.8% agarose gels.

**TABLE 1:** | Details of population locations, sample size, and genetic diversity of *V. japonicum* sampled in mainland China.

Code	Sampling locations	Longitude (°E)	Latitude (°N)	Altitude (m)	N	Num indv	Ho	He	π	FIS	s
DFS-1	Dongfushan Island, Zhoushan, Zhejiang Province, China	122***	30***	230.81	15	11.8013	0.2464	0.2831	0.2963	0.1412	0.2475
DFS-2		122***	30***	137.84	14	11.1198	0.2481	0.2895	0.3039	0.1512	0.2627
DC	Dachen Island, Jiaojiang, Taizhou, Zhejiang Province, China	121***	28***	101.49	13	9.9339	0.2073	0.2524	0.2666	0.1488	0.2591
TA	Tian’ao Island, Linhai Taizhou, Zhejiang Province, China	121***	28***	45.84	9	7.2946	0.1811	0.2128	0.2294	0.1175	0.2103
Mean							0.2207	0.2595	0.2741	0.1397	0.2452

N, sample size. Num Indv, average number of nucleotide differences. Ho, observed heterozygosity. He, expected heterozygosity. π, average nucleotide diversity. FIS, inbreeding coefficient within individuals. S, selfing rate. ***Because *V. japonicum* is endangered, some of the coordinates were hidden.

***Because *V. japonicum* is endangered, all of the coordinates were censored.

### 2.2 Library preparation and sequencing

The ddRAD-seq library construction was prepared by Shandong Expert Testing Technology Service Co. Ltd. (Qingdao, China). To achieve this, genomic DNA was digested with the suitable restriction enzyme *HindIII* and *MspI* at 37°C for 5 h. The resulting PCR products were purified, pooled, and electrophoresed on 2% agarose gels. DNA fragments ranging between 220 and 450 bps were isolated and purified, using the Agilent High Sensitivity DNA Kit. The DNA libraries were quantified using an Agilent Bioanalyzer. Finally, pools were combined in equimolar concentration to form a single genomic library and sequenced on an Illumina NovaSeq platform using 2 × 150 bp paired-end reads.

### 2.3 Quality filtering and SNP calling

The initial check of the raw reads was performed using FastQC v.0.11.9 (Andrews, 2010). To increase coverage depth and maximize loci, the *ustacks* function in Stacks software v 2.5.5 (Catchen, et al., 2013) was employed to identify ddRAD loci within individuals, requiring a minimum depth of four reads to form a stack and allow a maximum nucleotide mismatch between two stacks. To evaluate the effect of missing data on the analysis, a filtering parameter of *r* = 0.6 was employed, requiring≥ 60% of individuals in a population to process a locus. The raw SNPs data were further filtered using the following thresholds: (1) maximum observed heterozygosity of 0.75; (2) minor allele frequency (*MAF*) ≥ 0.02; and (3) minimum stack depth of *m* = 4 and *p* = 8 (i.e., one locus appeared in at least eight populations). Based on these calculations, a high-quality SNP dataset was obtained for further analysis.

### 2.4 Population genetic and statistical analyses

The genetic diversity parameters for each population including the average number of nucleotide differences (Num Indv), observed heterozygosity (*Ho*), expected heterozygosity (*He*), average nucleotide diversity (*π*), and inbreeding coefficient within individuals (*F*_*IS*_) were calculated using *Stacks* software v 2.5.5. The selfing rate (*s*) was calculated from *s* = 2*F*_*IS*_/(1 + *F*_*IS*_) (Barrière and Félix, 2005). To evaluate the differentiation within or among populations, analysis of molecular variance (AMOVA) and the genetic differentiation coefficient (*F*_*ST*_) were estimated using Arlequin v 3.5.2.2 (Excoffier, et al., 2005). To verify whether species fit the isolation-by-distance (IBD) patterns among populations, the relationship between pairwise genetic distance and geographic distance was evaluated by the Mantel test in Past software v 4.03 (Hammer, et al., 2001) with 9,999 random permutations. To determine the evolutionary relationship between populations, the phylogenetic tree was constructed based on the filtered SNPs using the Maximum Likelihood (ML) algorithm in the *FastTree* v 2.1 (Price, et al., 2010), and a rapid bootstrapping analysis with 1, 000 replications under the GTR + CAT model was conducted. Principal component analysis (PCA) was also used to evaluate the population structure and eigenvalues using the *GCTA* software v 1.94.1 (Yang, et al., 2011). The population structure of *V. japonicum* with different geographical distributions was visualized by the *ADMIXTURE* software v 1.3.0 (Alexander, et al., 2009) to cluster all samples with the ML algorithm. We conducted a *K*-means clustering analysis supposing the *K*-value ranged from 1 to 10. The clustering results were cross-validated and the most favorable fit for the number of clusters (*K*) value was determined by the cross-validation (*CV*) error. To further investigate the historical migration between four populations, an ML drift tree of populations and residual fitting heat map using *TreeMix* v1.3.1 (Pickrell and Pritchard, 2012) was also generated in this study. First, the ML tree was constructed, and then, migration arrows (events) were added to the tree sequentially, until a graph with the smallest residuals was found.

## 3 Results

### 3.1 SNPs identification by ddRAD-seq analysis

**FIGURE 2:**
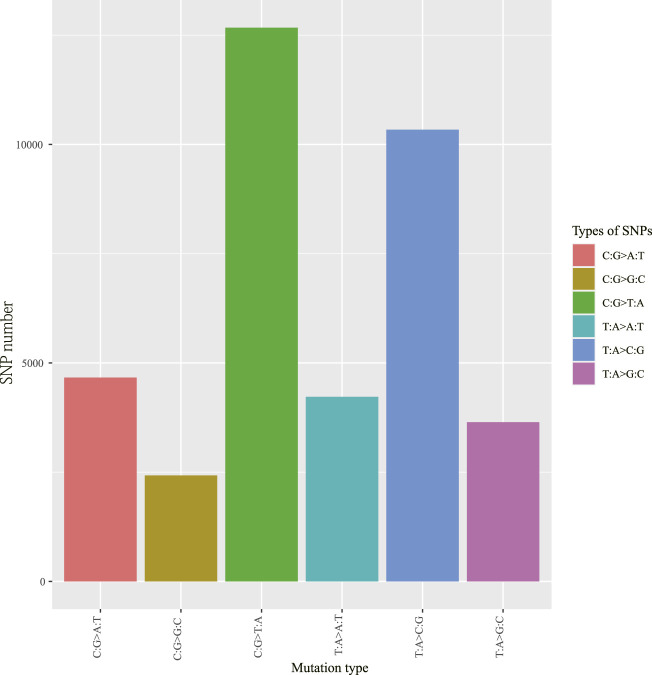
SNPs mutation types spectrum of *V. japonicum.*

To obtain high-resolution genotypic information on the 51 *V. japonicum* accessions, we utilized the ddRAD-seq method for SNPs genotyping. A total of 8,611,606 tags were acquired with an average sequence depth reaching 47.05 (range 34.48**–**64.32) ×. After filtering poor data, 445,060 SNPs were detected across the four populations. Among these, the majority were transitions (*Ts*) type SNPs (267,217), making up 60.04% of the total SNPs, while transversions (*Tv*) type SNPs (177,843) account for only 39.96%. The transitions to transversions ratio (*Ts*/*Tv*) was 1.503. Furthermore, heterozygosity SNPs (284,222) dominated, accounting for 63.86% of the total SNPs, whereas homozygosity SNPs (160,838) represented only 36.14%. Nevertheless, the distribution of SNP mutations was not uniform. The mutation spectrum analysis revealed that genome-wide SNP mutations could be divided into six types, with C:G > T:A and T:A > C:G as the main SNP mutants (Figure 2).

### 3.2 Genetic diversity and population divergence

Table 1 shows the genetic indices of the four *V. japonicum* populations. The highest level of average nucleotide diversity (*π*), observed heterozygosity (*Ho*) and expected heterozygosity (*He*) was recorded in population DFS-2 (*π* = 0.3039, *Ho =* 0.2481, *He =* 0.2895). By contrast, population TA exhibited the lowest diversity (*π* = 0.2294, *Ho =* 0.1811, *He =* 0.2128). Additionally, a lower observed heterozygosity (*Ho*) value was observed in all populations compared to expected heterozygosity (*He*), and, the *F*_*IS*_ values varied from 0.2130 to 0.2627, resulting in a mean selfing rate (*s*) of 24.52%.

At the species level, the AMOVA statistics of the 445,060 SNPs indicated significant genetic variation among (52.90%) and within (47.10%) the population (Table 2).

**TABLE 2:** Analysis of molecular variance (AMOVA) among and within populations of the studied *V. japonicum* individuals.

Source of variation	*df*	Sum of squares	Variance components	Percentage of variation (%)	*p*-value
Among populations	3	75822.532	1871.50524	52.90	<0.001
Within populations	47	78316.132	1666.30068	47.10	<0.001
Total	50	154138.664	3537.80592	100.00	<0.001

Furthermore, the average pairwise *F*_*ST*_ value was 0.1425 (range 0.0431–0.1924). DFS-2 population to TA had the highest level of differentiation compared to the remaining populations (Table 3).

**TABLE 3:** Genetic distance (*F*_*ST*_, above diagonal) and geographic distance (km, below diagonal) among four populations of *V. japonicum*.

	DFS-1	DFS-2	DC	TA
DFS-1	—	0.0431	0.1692	0.1917
DFS-2	0.616	—	0.1673	0.1924
DC	202.944	202.61	—	0.0912
TA	178.041	177.751	29.435	—

**FIGURE 3:**
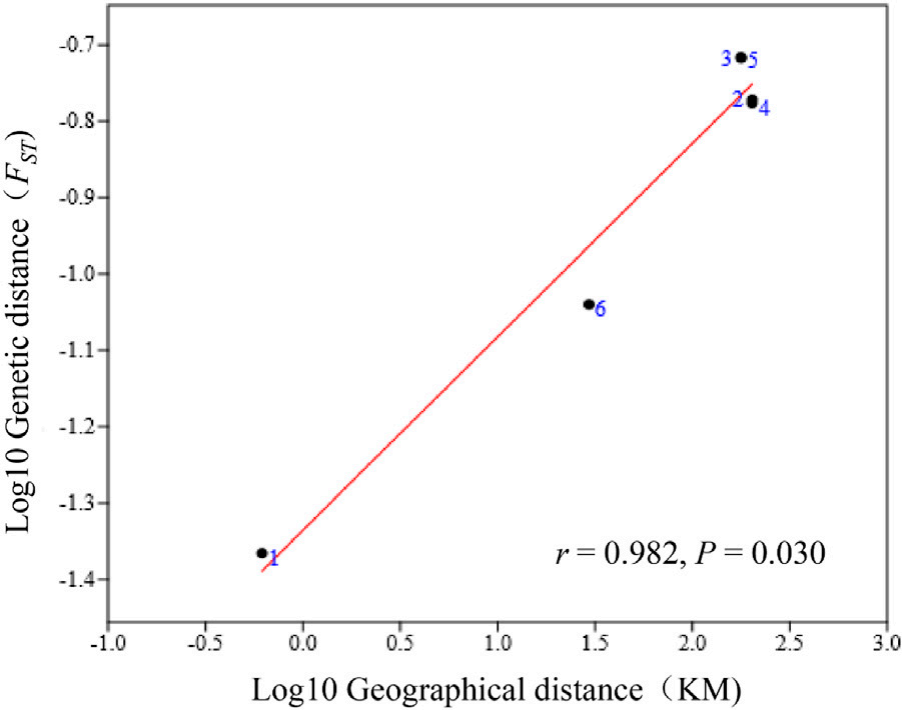
A Mantel test was conducted between the geographic and genetic distances among the four *V. japonicum* populations, using standardized data (log 10 genetic and log 10 geographic distances). The diagram displays dots marked with numbers that depict the six genetic and geographic distances generated from the combination of the four populations.

A significant positive correlation between genetic distances (pairwise *F*_*ST*_) and geographic distance (*r* = 0.982, *p* = 0.030) was detected by the Mantel test (Figure 3).

### 3.3 Genetic structure and historical migration

**FIGURE 4:**
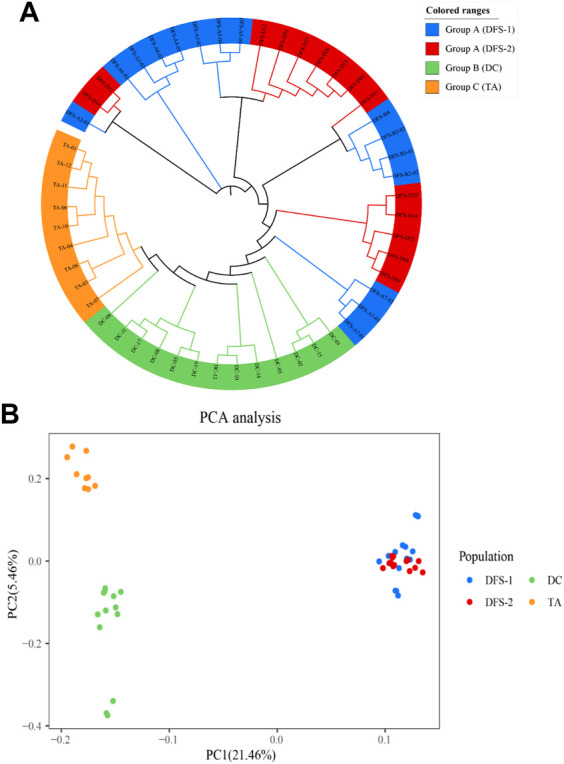
Phylogenetic relationship among the four *V. japonicum* populations was analyzed based on single nucleotide polymorphisms (SNPs). Shown through **(A)** the Maximum Likelihood (ML) tree and **(B)** scatter plots of the principal component analysis (PCA). Sampling location code are listed in Table 1.

According to the ML phylogenetic tree, the four populations were divided into three groups based on their genetic distance. Group A comprised individuals from DFS-1 and DFS-2. On the other hand, The individuals from DC and TA formed Group B and Group C, respectively (Figure 4A).

The contribution rates of the first principal component (PC1) and the second principal component (PC2) in the principal component analysis (PCA) were 21.46% and 5.46%, respectively. Furthermore, the PCA scatter plots of PCA reflected that DFS-1 and DFS-2 individuals were closely clustered together but distinct from individuals from DC and TA (Figure 4B).

**FIGURE 5:**
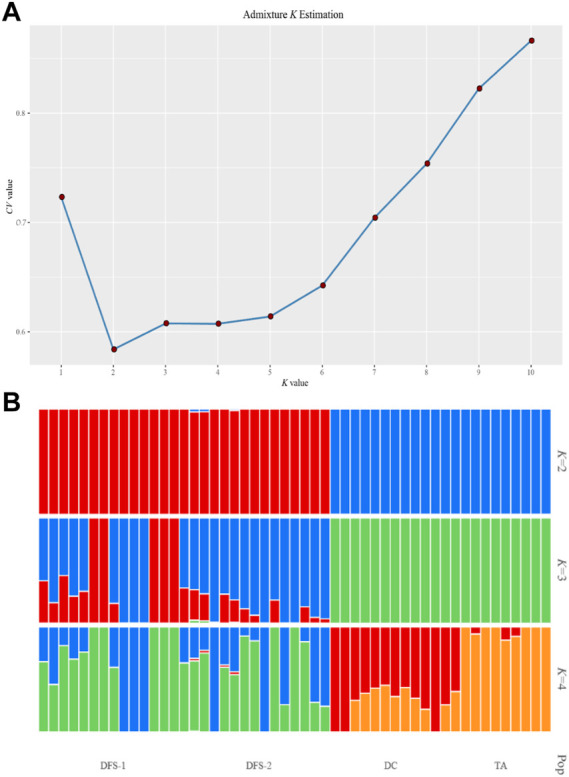
Population ADMIXTURE analysis of four *V. japonicum* populations based on single nucleotide polymorphisms (SNPs). **(A)** Possible *K* value estimation (*k* = 1–10) and **(B)** when the ancestor number is assumed to be 2–4. Each individual was represented by a vertical line, with its color indicating its proportion from ancestral populations.

The ADMIXTURE analysis for all SNPs showed that the optimum *K* value was 2 (Figure 5A), indicating that all 51 *V. japonicum* individuals evolved from two ancestral groups. Figure 5B showed that DC and TA evolved from one ancestor, while, DFS-1 and DFS-2 evolved from another ancestor.

**FIGURE 6:**
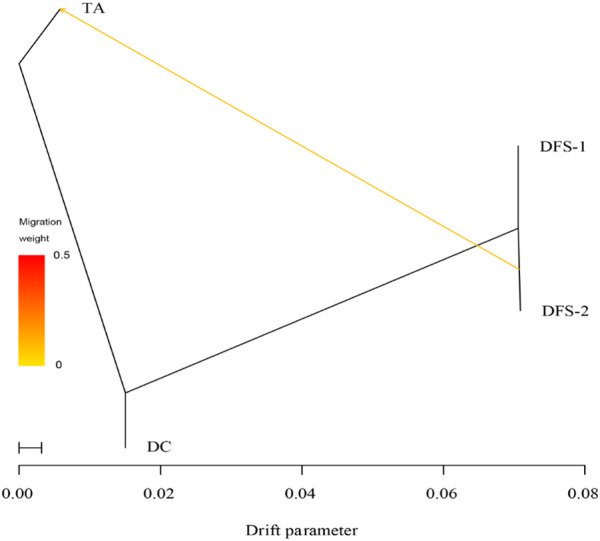
Patterns of historical gene flow using the TreeMix. The maximum-likelihood tree (ML) that best fits the data has one inferred migration event.

The TreeMix analysis supports one gene flow from DFS-2 to TA (Figure 6). Additionally, the heat map showed a value of 0 between all populations (Suppelementary Figure S1). This indicates that the ML tree model was accurately predicted and consistent with the inferred reality.

## 4 Discussion

### 4.1 Genetic diversity

Population genetic analysis is an essential tool in assessing the evolutionary potential and environmental adaptability of species and it is also considered one of the most important parameters to determine the priority in conservation genetic research (Wang, et al., 2021; Ji, et al., 2022). While sampling as many individuals as possible is desirable when studying population genetics, it can be challenging when investigating rare and endangered species, which often have a narrow range and lower genetic diversity than widely distributed plants (Hu, et al., 2022; Ji, et al., 2022). A recent study on the genetic diversity of *V. japonicum*, based on inter-sample sequence repeat (ISSR) revealed a high level of genetic diversity (Jiang, et al., 2021). However, different molecular markers can reveal different levels of genetic diversity (Zhang, et al., 2016). In contrast, analysis of SNPs showed that *V. japonicum* population maintained a moderate genetic diversity level (Table 1). The high density and uniform distribution of SNPs on the genome obtained by ddRAD-seq analysis (Janjua, et al., 2020), enable a more comprehensive and accurate picture of the genetic characteristics of *V. japonicum* than traditional molecular makers, providing a reference for future research.

The genetic diversity of species is influenced by several factors such as its distribution range, life history, breeding system, seed dispersal mechanism, and evolutionary history (Moritsuka, et al., 2017). *V. japonicum* similar to most *Viburnum* species, exhibits a low germination rate in the wild due to the presence of seed dormancy (Baskin, et al., 2009), which hinders the population’s development. Meanwhile, the natural distribution of *V. japonicum* is limited to islands, and while it can adapt to mild and moderate drought and light to some extent, long-term severe drought and high ultraviolet radiation prevalent on islands, can hamper its growth (Li, et al., 2018). This is likely to be a significant reason for the lower genetic diversity seen in its population when compared with other species. In addition, *V. japonicum* faces the dual impact of biological invasion and human activities, leading to severe damage to its habitats. Overall, narrow geographical distribution, shrinking habitats, and small population sizes are significant factors contributing to the reduction in its genetic diversity.

### 4.2 Genetic differentiation, gene flow and genetic structure

Several factors affect genetic differentiation among plant populations, with geographical isolation being a crucial factor that affects population growth, gene flow, and ultimately species’ persistence (Wu, et al., 2015). In this regard, natural islands provide the ideal laboratory for studying the geographical isolation of plants. In this study, we found a moderate level of genetic differentiation (average *F*_*ST*_ = 0.1425) between populations with the AMOVA analysis indicating that 52.9% of the genetic variation occurred across geographical regions. These results align well with the study findings of Jiang, et al. (2021), which used ISSR markers (52.71%). Furthermore, all populations had positive *F*_*IS*_ values, and the selfing rate (*s*) estimated was 24.52%, indicating that inbreeding occurred within all populations. The narrow geographical distribution and distance between the islands where *V. japonicum* grows can limit gene flow (pollen and seed) between populations, leading to increased genetic drift and inbreeding in the native population. These factors contribute to the increase of genetic differentiation observed among populations.

Notably, the distance between the TFS-2 and DC populations examined in this study was approximately 177.751 km, posing a significant geographic barrier hindering gene flow between these populations. However, one gene flow event was observed between the two populations in TreeMix. We calculated that *Nm* ≈ (1-*F*_*ST*_)/4*F*_*ST*_ = 0.046 < 1, indicating that gene flow between the two populations is limited. Nevertheless, field observations showed that *V. japonicum* has a high flowering capacity, displays bright red fruit, and produces an abundance of seeds. These traits attract local pollinators over short distances and a variety of frugivorous birds that can feed on the fruit and disperse seeds over long distances. Therefore, we hypothesize that sporadic birds dispersing seeds between islands facilitate the maintenance of gene flow between populations of *V. japonica*.

The Mantel test is a frequently used approach to evaluate spatial processes that influence population structure (Diniz-filho, et al., 2013). Our study utilized the Mantel test, which revealed a significant correlation between the genetic similarity and geographical location of *V. japonicum* (*r* = 0.982, *p* ≤ 0.030). This finding demonstrates that the geographic distance could be a limiting factor to gene flow between populations of *V. japonica*. The Mantel’s test results were further solidified through analysis of the ML tree, PCA, and ADMIXTURE.

### 4.3 Conservation and management strategies

Island plants tend to have lower genetic diversity than terrestrial species, and have a higher extinction risk (Pinheiro, et al., 2021). Human disturbance, rather than the limitations of pollen and seed dispersal, may be the direct cause of the population shrinkage and genetic diversity reduction of *V. japonica*, further exacerbated by the geographical isolation of islands which restricts the gene exchange between populations and intensifies the genetic differentiation among populations. Therefore, to enhance the protection of *V. japonica* and its utilization, it is recommended to strengthen the *in situ* protection of natural populations, regulate economic activities and artificial construction, such as wind turbines and solar power panels, in the vicinity of the populations, and prevent deforestation and reclamation to promote the population renewal and reproduction. Moreover, a germplasm resource bank can be established on Tian’ao Island, which can facilitate the mixing and breeding of individuals from different islands such as Dongfushan Island, Dachen Island, and other islands can be to promote gene exchange between populations and maximize the protection and restoration of the genetic diversity of *V. japonica*. Considering *V. japonica*’s specific habitat requirements, it would be beneficial to advance research on artificial breeding techniques to improve survival rates in ex-situ conservation. Additionally, by employing *in situ* population protection and ex-situ cultivation, excellent varieties can be chosen to support island vegetation restoration and landscaping design.

## Data Availability

The original contributions presented in the study are included in the article/Supplementary Material, further inquiries can be directed to the corresponding authors.
