# Supplementary figures and images for: Population genetic assessment of Viburnum japonicum in China using ddRAD-seq
Source: Front Genet. 2023 Jun 1;14:1150437. doi: 10.3389/fgene.2023.1150437 (PMC10267392; doi:10.3389/fgene.2023.1150437)

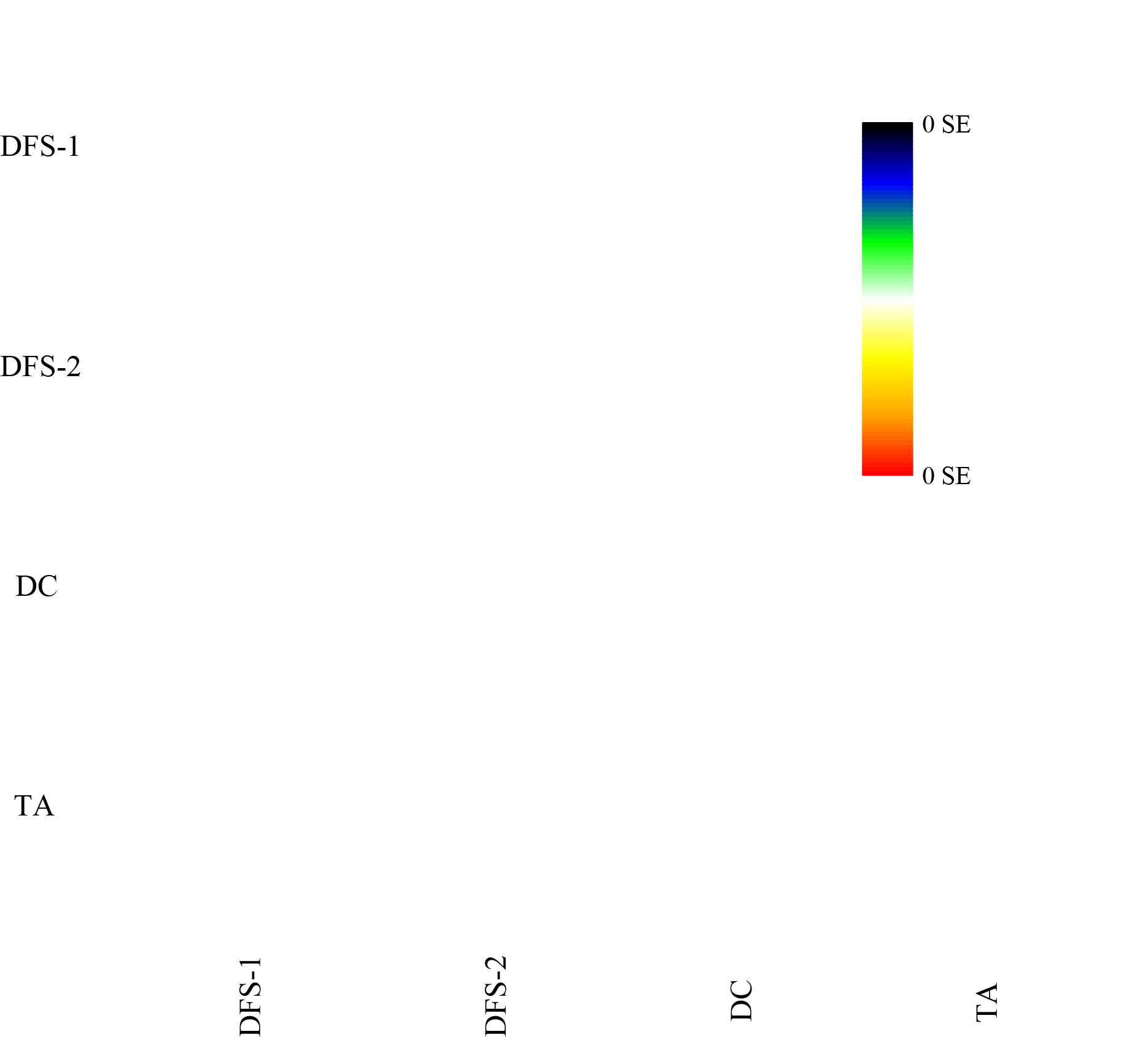

Supplement: Supplementary file 1 [file Image1.TIF]
